# Epidemiology and Prognostic Role of HPV Infection in Head and Neck Cancer: A Population‐Based Study of the SEER Database

**DOI:** 10.1002/cam4.71322

**Published:** 2025-11-21

**Authors:** Kangwen Guo, Jinmei Li, Haiyin Ye, Xiaoqiong Yi

**Affiliations:** ^1^ Department of Lymphoid Oncology Affiliated Hospital of Guangdong Medical University Zhanjiang China; ^2^ Department of Pulmonary Oncology Affiliated Hospital of Guangdong Medical University Zhanjiang China

**Keywords:** characteristics, head and neck cancer, HPV, prognosis and cox regression

## Abstract

**Background:**

Head and neck cancer (HNC) comprises a heterogeneous group of malignancies with significant variation in epidemiology, clinical features, and treatment responses. However, large‐scale data on the clinical epidemiology of HNC and the prognostic impact of human papillomavirus (HPV) infection have not yet been reported.

**Material and Methods:**

Data on HNC cases with known HPV status were obtained from the Surveillance, Epidemiology, and End Results (SEER) database. Clinical characteristics of HNC were summarized based on HPV status, primary site, and metastatic site. To elucidate the prognostic role of HPV status in HNC, we calculated the relative survival rate (RSR) and conducted Cox regression analysis following propensity score matching by HPV status.

**Results:**

A total of 14,855 HNC cases were included in this study, comprising 10,128 HPV‐positive and 4727 HPV‐negative cases. Clinical characteristics varied based on HPV status, primary site, and metastatic site. HPV positivity was generally associated with better prognosis, except in cases of nasopharyngeal carcinoma and cancers originating from the gum, as indicated by RSRs. Subgroup Cox regression analysis demonstrated significantly improved survival for HPV‐positive cases compared to their HPV‐negative counterparts across most categories.

**Conclusion:**

HNC encompasses a diverse group of heterogeneous diseases, with HPV positivity generally associated with a better prognosis.

## 1 Introduction

Head and neck cancer (HNC) encompasses a broad spectrum of heterogeneous cancer originating in the head and neck region, including cancers of the oral cavity, nasopharynx, oropharynx, larynx, hypopharynx, and other related sites, and each subtype within this group is associated with a unique etiology, epidemiological trends, and therapeutic approach [1]. Head and neck squamous cell carcinoma (HNSCC), the major histological type of HNC, is the sixth most common cancer worldwide [2, 3]. An estimated 59,600 new cases of cancer originating in the oral cavity and pharynx, along with 12,700 cancer‐related deaths, were estimated for 2025 [4]. According to estimates by the World Health Organization, 439,000 cases of mouth and oropharyngeal cancer are expected to occur in 2030 [5]. In terms of stage distribution, 29% of cases are classified as localized, 55% as regional, and 9% as distant in oral cavity and pharynx cancer [4]. Current HNC treatment options include surgery, radiotherapy, chemotherapy, targeted therapy, and immunotherapy, which vary on an individual basis and are normally determined by Multidisciplinary Team (MDT) discussion. For instance, surgeries represent a backbone for the treatment of early‐stage oral cavity cancers, whereas in advanced unresectable cases, chemoradiotherapy is preferred. Despite recent advancements like immunotherapy, long‐term survival remains low, with a 5‐year survival rate of 60.6% [5]. The five‐year survival rate for oral cavity and pharyngeal cancers varies by stage: 84% for localized cases, 65% for regional cases, and 39% for distant cases [6]. Survival disparities also exist among cancers originating from different sites, with 5‐year relative survival rates (RSRs) of 49.0% for the oral cavity, 54.8% for the oropharynx, 50.0% for the hypopharynx, and 63.4% for the larynx [5].

The epidemiological trend of HNC has shifted significantly due to the rising incidence of human papillomavirus (HPV)‐associated oropharyngeal cancer [7, 8]. In the USA, approximately 25,000 HPV‐related cancers are diagnosed annually, with HPV infection accounting for 90% of anal cancers and 65% of vaginal cancers [2, 9, 10, 11]. HPV has been detected in 23%–35% of HNSCC cases globally, with most arising from the oropharynx, where HPV positivity rates range between 45% and 90% [11, 12, 13]. While HPV has been well established as a driver of oropharyngeal cancer, its role in other HNC remains controversial. Interestingly, HPV‐positive and HPV‐negative diseases represent two distinct groups, differing in epidemiology, clinicopathological characteristics, and treatment response [14, 15]. HPV‐associated HNC generally presents with a limited T stage, a more advanced N stage, and an overall advanced stage [16]. Patients with HPV‐associated cases are typically younger, non‐smokers, male, of higher socioeconomic status, and Caucasian [17, 18, 19, 20]. A recent radiomic study indicates that HPV‐negative cancers are radiologically more aggressive, showing signs of invasion into the prevertebral fascia, posterior wall of the hypopharynx, and other structures [21]. Additionally, HPV‐related cases are associated with fewer comorbidities and greater sensitivity to chemotherapy and radiotherapy [1].

In the era of precision medicine, it is crucial to investigate the impact of HPV infection on HNC prognosis and to analyze its epidemiological trends using larger sample sizes. Such studies may provide valuable insights for bench research and clinical management. However, most previous studies relied on small samples rather than population‐based data, limiting their representativeness and generalizability. Additionally, regarding the metastatic patterns of HNC, prior research has primarily focused on metachronous metastasis, which develops during post‐treatment follow‐up [22, 23, 24, 25, 26, 27]. We hypothesize that a study based on a population‐based dataset may provide more solid evidence about revealing HNC epidemiology. This study aims to elaborate on the role of HPV in HNC prognosis using data from the Surveillance, Epidemiology, and End Results (SEER) database.

## 2 Materials and Methods

### 2.1 Case Inclusion

All cases in the current study were obtained from the SEER cancer registry, established by the National Cancer Institute in 1973 with the original nine registries: Connecticut, Detroit, Atlanta, San Francisco‐Oakland, Hawaii, Iowa, New Mexico, Seattle‐Puget Sound, and Utah. The registry later expanded to include 13 and subsequently 18 registries. The SEER database records clinicopathological and demographic information, including sex, age, year of diagnosis, tumor stage, survival time, survival status, and other relevant details [28]. Currently, the 18 SEER registries cover approximately 28% of the total U.S. population, representing the most comprehensive data resource for cancer epidemiological studies [29]. Data collection was performed using SEER*Stat version 8.3.2 [30]. Since HPV status first became available in 2010, only cases diagnosed after January 1, 2010, with definitive HPV status were included in this study. Multiple methodologies exist for ascertaining HPV status in the database. This field is reserved for HPV status confirmed through molecular techniques such as in situ hybridization (ISH), polymerase chain reaction (PCR), or reverse transcription PCR (RT‐PCR), which directly identify viral DNA or RNA, instead of surrogate markers like p16 [31]. To enhance study quality, only cases with active and complete follow‐up were enrolled. Cases diagnosed solely by autopsy or death certificate were excluded, and only cases confirmed by positive histology were included. All cases included were staged based on the AJCC seventh edition. Age was categorized into four groups: 0–45, 46–60, 61–75, and 75+. Race and tumor grade classifications adhered to the standardized definitions established by the SEER database, ensuring consistency with nationally recognized criteria [29]. Based on ICD‐O‐3 site codes, the primary regions were grouped as follows: gum and other mouth, hypopharynx, nasopharynx, oropharynx, other oral cavity and pharynx, tongue, and tonsil [32]. The following Histologic Type ICD‐O‐3 were used to identify solid tumors in the head and neck region (C000‐C148, C300‐C329, C410, C411, C442). Case ascertainment aligned with the SEER coding guidelines to ensure standardized tumor classification. The patient selection protocol was algorithmically executed within the SEER*Stat platform with predefined inclusion/exclusion criteria, consistent with established methodologies for SEER database analyses. Subsequent to data retrieval, the analytical exclusion involved implementation of propensity score matching, yielding a balanced cohort of 7100 cases (3550 matched pairs) derived from an initial population of 14,855 HNC cases.

### 2.2 Statistical Analyses

To clarify the impact of HPV infection on the prognosis of HNC patients, we calculated the RSRs, which are widely used to illustrate net survival directly attributable to the index cancer [33, 34, 35, 36, 37, 38, 39, 40]. RSR was calculated using the Ederer II methodology and the expected survival table: U.S. 1970–2015 by individual year (Caucasian, African American, Other [AI/API], Ages 0–99, and all races for Other Unspecified 1991+ and Unknown). Specifically, RSRs were determined by dividing the observed survival (percentage of HNC patients alive at a given time point) by the expected survival (percentage of the general population alive at the same time point). Differences in all‐cause survival were evaluated using the Kaplan–Meier method with log‐rank tests for comparison. To improve comparability between HPV‐positive and HPV‐negative patients, we performed propensity score matching based on HPV status using the nearest neighbor method with a caliper of 0.01 and a ratio of 1:1. Adjustments were made for age group, sex, year of diagnosis, race, primary site, grade, surgery, radiation, chemotherapy, insurance status, marital status, and T, N, and M stages [41]. For survival analysis, the observation period spanned from the date of diagnosis to December 2021, with administrative censoring applied at the study endpoint to ensure uniform follow‐up. The risk factors for all‐cause mortality were assessed using univariate Cox regression and subsequently multivariate Cox regression, with respective 95% confidence intervals for both regression analyses [42]. Only risk factors demonstrating statistically significant prognostic value in univariate Cox regression were incorporated into the multivariate analysis. To validate the proportional hazards assumption essential for Cox regression modeling, the Schoenfeld residuals test was performed by the survival package in R. The results confirmed no significant violations of the proportional hazards assumption, supporting the appropriateness of the Cox model for this analysis. The statistical tests include the *t*‐test or Mann–Whitney *U* test, as appropriate, for continuous variables; ANOVA for multi‐group comparisons of continuous variables; and Cox regression for survival modeling. All statistical analyses were conducted using the *survival* and *MatchIt* packages in R version 3.3.3 [43, 44, 45]. A two‐tailed *p* value less than 0.05 was defined as significant.

## 3 Results

### 3.1 Baseline Characteristics

A total of 14,855 HNC cases were included in this study, comprising 10,128 HPV‐positive and 4727 HPV‐negative cases (Table 1). Significant differences were observed between HPV‐positive and HPV‐negative patients in terms of sex, age, race, year of diagnosis, site, differentiation level, surgery, radiotherapy, chemotherapy, insurance status, marital status, lung metastasis, brain metastasis, liver metastasis, bone metastasis, T stage, N stage, and M stage (*p* < 0.001). After propensity score matching at a 1:1 ratio, 3550 HPV‐positive and 3550 HPV‐negative patients were matched, with no significant differences in the aforementioned clinicopathological factors (Table 1).

**TABLE 1:** The baseline characteristics of head and neck cancer by HPV status of all enrolled cases or matched cases by propensity score matching.

Categories	All cases	Original data			Matched data		
		HPV (+) cases	HPV (−) cases	*p*	HPV (+) cases	HPV (−) cases	*p*
All cases	14,855	10,128	4727		3550	3550	
Sex				< 0.001			> 0.05
Male	12,364 (83.2)	8770 (86.6)	3594 (76.0)		2805 (79.0)	2829 (79.7)	
Age				< 0.001			> 0.05
Mean (SD)	53.93 (10.28)	53.54 (9.6)	54.77 (11.54)		54.28 (10.76)	54.42 (10.82)	
0–45	2779 (18.7)	1917 (18.9)	862 (18.2)		690 (19.4)	645 (18.2)	
46–60	8479 (57.1)	6007 (59.3)	2472 (52.3)		1887 (53.2)	1943 (54.7)	
61–75	3236 (21.8)	2035 (20.1)	1201 (25.4)		865 (24.4)	858 (24.2)	
75+	361 (2.4)	169 (1.7)	192 (4.1)		108 (3.0)	104 (2.9)	
Race				< 0.001			> 0.05
White	12,682 (85.4)	9073 (89.6)	3609 (76.3)		2890 (81.4)	2909 (81.9)	
AI	121 (0.8)	73 (0.7)	48 (1.0)		38 (1.1)	32 (0.9)	
API	760 (5.1)	336 (3.3)	424 (9.0)		222 (6.3)	208 (5.9)	
AA	1189 (8.0)	571 (5.6)	618 (13.1)		375 (10.6)	375 (10.6)	
Unknown	103 (0.7)	75 (0.7)	28 (0.6)		25 (0.7)	26 (0.7)	
Year of diagnosis				< 0.001			> 0.05
2010	901 (6.1)	557 (5.5)	344 (7.3)		259 (7.3)	257 (7.2)	
2011	1414 (9.5)	827 (8.2)	587 (12.4)		402 (11.3)	411 (11.6)	
2012	1788 (12.0)	1127 (11.1)	661 (14.0)		474 (13.4)	470 (13.2)	
2013	2240 (15.1)	1510 (14.9)	730 (15.4)		549 (15.5)	547 (15.4)	
2014	2585 (17.4)	1794 (17.7)	791 (16.7)		587 (16.5)	607 (17.1)	
2015	2765 (18.6)	2005 (19.8)	760 (16.1)		593 (16.7)	583 (16.4)	
2016	3162 (21.3)	2308 (22.8)	854 (18.1)		686 (19.3)	675 (19.0)	
Site				< 0.001			> 0.05
Gum and other mouth	307 (2.1)	64 (0.6)	243 (5.1)		64 (1.8)	84 (2.4)	
Hypopharynx	811 (5.5)	204 (2.0)	607 (12.8)		201 (5.7)	224 (6.3)	
Nasopharynx	890 (6.0)	285 (2.8)	605 (12.8)		278 (7.8)	278 (7.8)	
Oropharynx	955 (6.4)	543 (5.4)	412 (8.7)		330 (9.3)	302 (8.5)	
Other oral cavity and pharynx	307 (2.1)	157 (1.6)	150 (3.2)		104 (2.9)	112 (3.2)	
Tongue	4935 (33.2)	3662 (36.2)	1273 (26.9)		1139 (32.1)	1180 (33.2)	
Tonsil	6650 (44.8)	5213 (51.5)	1437 (30.4)		1434 (40.4)	1370 (38.6)	
Differentiation				< 0.001			> 0.05
Grade I	452 (3.0)	213 (2.1)	239 (5.1)		152 (4.3)	143 (4.0)	
Grade II	4254 (28.6)	2589 (25.6)	1665 (35.2)		1174 (33.1)	1204 (33.9)	
Grade III	6033 (40.6)	4422 (43.7)	1611 (34.1)		1283 (36.1)	1299 (36.6)	
Grade IV	370 (2.5)	183 (1.8)	187 (4.0)		107 (3.0)	98 (2.8)	
Unknown	3746 (25.2)	2721 (26.9)	1025 (21.7)		834 (23.5)	806 (22.7)	
Surgery				< 0.001			> 0.05
Surgery	5047 (34.0)	3773 (37.3)	1274 (27.0)		1111 (31.3)	1066 (30.0)	
Tumor destruction	10 (0.1)	5 (0.0)	5 (0.1)		4 (0.1)	5 (0.1)	
No	9765 (65.7)	6330 (62.5)	3435 (72.7)		2422 (68.2)	2470 (69.6)	
Unknown	33 (0.2)	20 (0.2)	13 (0.3)		13 (0.4)	9 (0.3)	
Radiotherapy				< 0.001			> 0.05
Yes	12,685 (85.4)	8901 (87.9)	3784 (80.1)		2894 (81.5)	2935 (82.7)	
Refused	176 (1.2)	123 (1.2)	53 (1.1)		57 (1.6)	39 (1.1)	
Unknown	1994 (13.4)	1104 (10.9)	890 (18.8)		599 (16.9)	576 (16.2)	
Chemotherapy				< 0.001			> 0.05
Yes	10,560 (71.1)	7325 (72.3)	3235 (68.4)		1131 (31.9)	1063 (29.9)	
Insurance				< 0.001			> 0.05
Insured	14,049 (94.6)	9678 (95.6)	4371 (92.5)		3301 (93.0)	3311 (93.3)	
Uninsured	529 (3.6)	290 (2.9)	239 (5.1)		162 (4.6)	156 (4.4)	
Unknown	277 (1.9)	160 (1.6)	117 (2.5)		87 (2.5)	83 (2.3)	
Marriage				< 0.001			> 0.05
Married	8539 (57.5)	6221 (61.4)	2318 (49.0)		1780 (50.1)	1876 (52.8)	
Unmarried	5544 (37.3)	3414 (33.7)	2130 (45.1)		1548 (43.6)	1473 (41.5)	
Unknown	772 (5.2)	493 (4.9)	279 (5.9)		222 (6.3)	201 (5.7)	
Lung metastasis				< 0.001			> 0.05
NA	2 (0.0)	0 (0.0)	2 (0.0)		0 (0.0)	1 (0.0)	
No	14,405 (97.0)	9905 (97.8)	4500 (95.2)		3407 (96.0)	3415 (96.2)	
Unknown	162 (1.1)	96 (0.9)	66 (1.4)		60 (1.7)	44 (1.2)	
Yes	286 (1.9)	127 (1.3)	159 (3.4)		83 (2.3)	90 (2.5)	
Brain metastasis				< 0.001			> 0.05
NA	2 (0.0)	0	2 (0.0)		0 (0.0)	1 (0.0)	
No	14,681 (98.8)	10,036 (99.1)	4645 (98.3)		3490 (98.3)	3493 (98.4)	
Unknown	144 (1.0)	83 (0.8)	61 (1.3)		52 (1.5)	43 (1.2)	
Yes	28 (0.2)	9 (0.1)	19 (0.4)		8 (0.2)	13 (0.4)	
Liver metastasis				< 0.001			> 0.05
NA	2 (0.0)	0	2 (0.0)		0 (0.0)	1 (0.0)	
No	14,591 (98.2)	9989 (98.6)	4602 (97.4)		3459 (97.4)	3473 (97.8)	
Unknown	140 (0.9)	83 (0.8)	57 (1.2)		51 (1.4)	39 (1.1)	
Yes	122 (0.8)	56 (0.6)	66 (1.4)		40 (1.1)	37 (1.0)	
Bone metastasis				< 0.001			> 0.05
NA	3 (0.0)	1 (0.0)	2 (0.0)		0 (0.0)	1 (0.0)	
No	14,512 (97.7)	9957 (98.3)	4555 (96.4)		3443 (97.0)	3439 (96.9)	
Unknown	143 (1.0)	79 (0.8)	64 (1.4)		49 (1.4)	44 (1.2)	
Yes	197 (1.3)	91 (0.9)	106 (2.2)		58 (1.6)	66 (1.9)	
T stage				< 0.001			> 0.05
0	64 (0.4)	50 (0.5)	14 (0.3)		16 (0.5)	10 (0.3)	
1	3502 (23.6)	2582 (25.5)	920 (19.5)		710 (20.0)	727 (20.5)	
2	4844 (32.6)	3557 (35.1)	1287 (27.2)		1014 (28.6)	1017 (28.6)	
3	2473 (16.6)	1568 (15.5)	905 (19.1)		662 (18.6)	651 (18.3)	
4	2320 (15.6)	1327 (13.1)	993 (21.0)		674 (19.0)	677 (19.1)	
NA	365 (2.5)	189 (1.9)	176 (3.7)		120 (3.4)	131 (3.7)	
TX	1287 (8.7)	855 (8.4)	432 (9.1)		354 (10.0)	337 (9.5)	
N stage				< 0.001			> 0.05
0	2366 (15.9)	1311 (12.9)	1055 (22.3)		683 (19.2)	664 (18.7)	
1	2701 (18.2)	1755 (17.3)	946 (20.0)		758 (21.4)	703 (19.8)	
2	8505 (57.3)	6310 (62.3)	2195 (46.4)		1728 (48.7)	1814 (51.1)	
3	767 (5.2)	477 (4.7)	290 (6.1)		211 (5.9)	196 (5.5)	
NA	365 (2.5)	189 (1.9)	176 (3.7)		120 (3.4)	131 (3.7)	
NX	151 (1.0)	86 (0.8)	65 (1.4)		50 (1.4)	42 (1.2)	
M stage				< 0.001			> 0.05
0	13,894 (93.5)	9643 (95.2)	4251 (89.9)		3242 (91.3)	3240 (91.3)	
1	596 (4.0)	296 (2.9)	300 (6.3)		188 (5.3)	179 (5.0)	
NA	365 (2.5)	189 (1.9)	176 (3.7)		120 (3.4)	131 (3.7)	

Abbreviations: AA, African American; AI, American Indian/Alaska Native; API, Asian or Pacific Islander; NA, non‐applicable; NX, unknown N stage; SD, standard deviation; TX, unknown T stage.

Baseline characteristics by primary site were also summarized (Table 2). Surgery was more prevalent in cancers originating from the tonsil (48.7%) and gum and other mouth (40.4%) compared to other HNC types. Radiotherapy was widely adopted for HNC management, with rates ranging from 65.5% to 87.9%. Notably, nasopharyngeal cancer distinguished itself with the youngest mean age at diagnosis (48 years), higher prevalence among Asian or Pacific Islanders, and a higher incidence of lung, liver, and bone metastases at diagnosis. Cancers originating from the gum and other mouth were characterized by a higher percentage of female cases, greater prevalence among African American patients, and a higher incidence of N0 stage cases.

**TABLE 2:** The baseline characteristics of head and neck cancer by originated site.

Categories	Originated site							*p*
	Tonsil	Gum and other mouth	Hypopharynx	Nasopharynx	Oropharynx	Other oral cavity and pharynx	Tongue	
All cases	6650	307	811	890	955	307	4935	
HPV status								< 0.001
Positive	5213 (78.4)	64 (20.8)	204 (25.2)	285 (32)	543 (56.9)	157 (51.1)	3662 (74.2)	
Sex								
Male	5568 (83.7)	202 (65.8)	660 (81.4)	627 (70.4)	770 (80.6)	255 (83.1)	4282 (86.8)	< 0.001
Age								
Mean (SD)	52.72 (9.57)	56.57 (10.82)	57.2 (10.06)	48 (14.27)	54.84 (10.19)	55.26 (10.44)	55.68 (9.66)	< 0.001
0–45	1452 (21.8)	42 (13.7)	86 (10.6)	352 (39.6)	147 (15.4)	44 (14.3)	656 (13.3)	< 0.001
46–60	3907 (58.8)	165 (53.7)	430 (53.0)	377 (42.4)	546 (57.2)	176 (57.3)	2878 (58.3)	
61–75	1180 (17.7)	83 (27)	261 (32.2)	144 (16.2)	233 (24.4)	77 (25.1)	1258 (25.5)	
75+	111 (1.7)	17 (5.5)	34 (4.2)	17 (1.9)	29 (3.0)	10 (3.3)	143 (2.9)	
Race								< 0.001
White	5846 (87.9)	237 (77.2)	640 (78.9)	421 (47.3)	793 (83)	277 (90.2)	4468 (90.5)	
AI	45 (0.7)	1 (0.3)	12 (1.5)	21 (2.4)	8 (0.8)	1 (0.3)	33 (0.7)	
API	208 (3.1)	12 (3.9)	53 (6.5)	335 (37.6)	28 (2.9)	6 (2)	118 (2.4)	
AA	494 (7.4)	53 (17.3)	106 (13.1)	101 (11.3)	117 (12.3)	23 (7.5)	295 (6)	
Unknown	57 (0.9)	4 (1.3)	0 (0)	12 (1.3)	9 (0.9)	0 (0)	21 (0.4)	
Year of diagnosis								0.062
2010	428 (6.4)	16 (5.2)	46 (5.7)	57 (6.4)	57 (6)	15 (4.9)	282 (5.7)	
2011	642 (9.7)	23 (7.5)	69 (8.5)	99 (11.1)	82 (8.6)	36 (11.7)	463 (9.4)	
2012	819 (12.3)	41 (13.4)	113 (13.9)	99 (11.1)	97 (10.2)	23 (7.5)	596 (12.1)	
2013	1027 (15.4)	44 (14.3)	123 (15.2)	137 (15.4)	148 (15.5)	47 (15.3)	714 (14.5)	
2014	1153 (17.3)	60 (19.5)	137 (16.9)	116 (13)	180 (18.8)	57 (18.6)	882 (17.9)	
2015	1215 (18.3)	58 (18.9)	140 (17.3)	185 (20.8)	173 (18.1)	50 (16.3)	944 (19.1)	
2016	1366 (20.5)	65 (21.2)	183 (22.6)	197 (22.1)	218 (22.8)	79 (25.7)	1054 (21.4)	
Differentiation								< 0.001
Grade I	182 (2.7)	30 (9.8)	30 (3.7)	10 (1.1)	44 (4.6)	10 (3.3)	146 (3)	
Grade II	2004 (30.1)	137 (44.6)	311 (38.3)	74 (8.3)	308 (32.3)	68 (22.1)	1352 (27.4)	
Grade III	2960 (44.5)	85 (27.7)	285 (35.1)	311 (34.9)	294 (30.8)	55 (17.9)	2043 (41.4)	
Grade IV	80 (1.2)	3 (1)	8 (1)	209 (23.5)	11 (1.2)	2 (0.7)	57 (1.2)	
Unknown	1424 (21.4)	52 (16.9)	177 (21.8)	286 (32.1)	298 (31.2)	172 (56)	1337 (27.1)	
Surgery								< 0.001
Surgery	3241 (48.7)	124 (40.4)	153 (18.9)	83 (9.3)	249 (26.1)	53 (17.3)	1144 (23.2)	
Tumor destruction	4 (0.1)	1 (0.3)	1 (0.1)	0 (0)	0 (0.0)	1 (0.3)	3 (0.1)	
No	3392 (51.0)	179 (58.3)	655 (80.8)	807 (90.7)	704 (73.7)	250 (81.4)	3778 (76.6)	
Unknown	13 (0.2)	3 (1)	2 (0.2)	0 (0)	2 (0.2)	3 (1.0)	10 (0.2)	
Radiotherapy								< 0.001
Yes	5738 (86.3)	201 (65.5)	657 (81)	782 (87.9)	775 (81.2)	231 (75.2)	4301 (87.2)	
Refused	66 (1)	6 (2)	9 (1.1)	5 (0.6)	16 (1.7)	1 (0.3)	73 (1.5)	
Unknown	846 (12.7)	100 (32.6)	145 (17.9)	103 (11.6)	164 (17.2)	75 (24.4)	561 (11.4)	
Chemotherapy								< 0.001
No/unknown	2051 (30.8)	168 (54.7)	229 (28.2)	136 (15.3)	296 (31)	136 (44.3)	1279 (25.9)	
Insurance								< 0.001
Insured	6274 (94.3)	286 (93.2)	763 (94.1)	829 (93.1)	883 (92.5)	286 (93.2)	4728 (95.8)	
Uninsured	237 (3.6)	12 (3.9)	35 (4.3)	45 (5.1)	49 (5.1)	14 (4.6)	137 (2.8)	
Unknown	139 (2.1)	9 (2.9)	13 (1.6)	16 (1.8)	23 (2.4)	7 (2.3)	70 (1.4)	
Marriage								< 0.001
Married	3903 (58.7)	130 (42.3)	355 (43.8)	508 (57.1)	454 (47.5)	160 (52.1)	3029 (61.4)	
Unmarried	2394 (36)	152 (49.5)	403 (49.7)	336 (37.8)	434 (45.4)	132 (43)	1693 (34.3)	
Unknown	353 (5.3)	25 (8.1)	53 (6.5)	46 (5.2)	67 (7)	15 (4.9)	213 (4.3)	
Lung metastasis								< 0.001
NA	0 (0)	0 (0)	0 (0)	0 (0)	0 (0)	2 (0.7)	0 (0)	
No	6511 (97.9)	295 (96.1)	769 (94.8)	841 (94.5)	908 (95.1)	286 (93.2)	4795 (97.2)	
Unknown	59 (0.9)	5 (1.6)	9 (1.1)	15 (1.7)	16 (1.7)	14 (4.6)	44 (0.9)	
Yes	80 (1.2)	7 (2.3)	33 (4.1)	34 (3.8)	31 (3.2)	5 (1.6)	96 (1.9)	
Brain metastasis								< 0.001
NA	0 (0)	0 (0)	0 (0)	0 (0)	0 (0)	2 (0.7)	0 (0)	
No	6585 (99)	302 (98.4)	799 (98.5)	868 (97.5)	937 (98.1)	289 (94.1)	4901 (99.3)	
Unknown	56 (0.8)	5 (1.6)	10 (1.2)	12 (1.3)	15 (1.6)	14 (4.6)	32 (0.6)	
Yes	9 (0.1)	0 (0)	2 (0.2)	10 (1.1)	3 (0.3)	2 (0.7)	2 (0)	
Liver metastasis								< 0.001
NA	0 (0)	0 (0)	0 (0)	0 (0)	0 (0)	2 (0.7)	0 (0)	
No	6566 (98.7)	299 (97.4)	793 (97.8)	851 (95.6)	929 (97.3)	286 (93.2)	4867 (98.6)	
Unknown	56 (0.8)	4 (1.3)	9 (1.1)	11 (1.2)	15 (1.6)	13 (4.2)	32 (0.6)	
Yes	28 (0.4)	4 (1.3)	9 (1.1)	28 (3.1)	11 (1.2)	6 (2)	36 (0.7)	
Bone metastasis								< 0.001
NA	0 (0)	0 (0)	0 (0)	0 (0)	0 (0)	3 (1)	0 (0)	
No	6535 (98.3)	301 (98)	784 (96.7)	831 (93.4)	929 (97.3)	283 (92.2)	4849 (98.3)	
Unknown	56 (0.8)	4 (1.3)	16 (2)	46 (5.2)	14 (1.5)	15 (4.9)	30 (0.6)	
Yes	59 (0.9)	2 (0.7)	11 (1.4)	13 (1.5)	12 (1.3)	6 (2)	56 (1.1)	
T stage								< 0.001
0	5 (0.1)	0 (0)	1 (0.1)	6 (0.7)	42 (4.4)	0 (0)	10 (0.2)	
1	1774 (26.7)	92 (30)	79 (9.7)	267 (30)	137 (14.3)	0 (0)	1153 (23.4)	
2	2522 (37.9)	86 (28)	262 (32.3)	166 (18.7)	183 (19.2)	0 (0)	1625 (32.9)	
3	989 (14.9)	49 (16)	184 (22.7)	181 (20.3)	176 (18.4)	0 (0)	894 (18.1)	
4	787 (11.8)	54 (17.6)	209 (25.8)	222 (24.9)	261 (27.3)	0 (0)	787 (15.9)	
NA	20 (0.3)	4 (1.3)	3 (0.4)	10 (1.1)	6 (0.6)	307 (100)	15 (0.3)	
TX	553 (8.3)	22 (7.2)	73 (9)	38 (4.3)	150 (15.7)	0 (0)	451 (9.1)	
N stage								< 0.001
0	1029 (15.5)	149 (48.5)	204 (25.2)	178 (20)	180 (18.8)	0 (0)	626 (12.7)	
1	1239 (18.6)	43 (14)	135 (16.6)	296 (33.3)	157 (16.4)	0 (0)	831 (16.8)	
2	3981 (59.9)	96 (31.3)	411 (50.7)	273 (30.7)	547 (57.3)	0 (0)	3197 (64.8)	
3	306 (4.6)	11 (3.6)	48 (5.9)	120 (13.5)	53 (5.5)	0 (0)	229 (4.6)	
NA	20 (0.3)	4 (1.3)	3 (0.4)	10 (1.1)	6 (0.6)	307 (100)	15 (0.3)	
NX	75 (1.1)	4 (1.3)	10 (1.2)	13 (1.5)	12 (1.3)	0 (0)	37 (0.7)	
M stage								< 0.001
0	6436 (96.8)	288 (93.8)	753 (92.8)	792 (89)	895 (93.7)	0 (0)	4730 (95.8)	
1	194 (2.9)	15 (4.9)	55 (6.8)	88 (9.9)	54 (5.7)	0 (0)	190 (3.9)	
NA	20 (0.3)	4 (1.3)	3 (0.4)	10 (1.1)	6 (0.6)	307 (100)	15 (0.3)	

Abbreviations: AA, African American; AI, American Indian/Alaska Native; API, Asian or Pacific Islander; NA, non‐applicable; NX, unknown N stage; SD, standard deviation; TX, unknown T stage.

Regarding the metastatic patterns of HNC, lung, liver, and bone were the most common sites for metastasis, with crude incidences of 2%, 1%, and 1%, respectively (Table 3). Female HNC patients exhibited a higher propensity for metastasis, particularly to the bone and lung, compared to males. Asian and Pacific Islanders had the highest risk of metastatic disease, including bone, brain, liver, and lung metastases. Additionally, African American patients were found to have a higher risk of lung metastasis (4%).

**TABLE 3:** The baseline characteristics of head and neck cancer by metastatic site at diagnosis.

Categories	Bone metastasis			Brain metastasis			Liver metastasis			Lung metastasis		
	No	Yes	*p*	No	Yes	*p*	No	Yes	*p*	No	Yes	*p*
*N*	14,512 (98.66)	197 (1.34)		14,681 (99.81)	28 (0.19)		14,591 (99.17)	122 (0.83)		14,405 (98.05)	286 (1.95)	
Sex			0.505			0.922			1			0.031
Male	12,083 (98.69)	160 (1.31)		12,219 (99.8)	24 (0.2)		12,144 (99.17)	102 (0.83)		12,002 (98.17)	224 (1.83)	
Female	2429 (98.5)	37 (1.5)		2462 (99.84)	4 (0.16)		2447 (99.19)	20 (0.81)		2403 (97.48)	62 (2.52)	
Age												
Mean (SD)	53.92 (10.22)	53.23 (12.76)	0.088	53.92 (10.26)	49.86 (11.27)	0.008	53.91 (10.26)	53.80 (10.28)	0.032	53.88 (10.25)	55.28 (10.50)	< 0.001
Age group			0.002			0.005			0.008			< 0.001
0–45	2716 (98.55)	40 (1.45)		2751 (99.82)	5 (0.18)		2737 (99.2)	22 (0.8)		2715 (98.55)	40 (1.45)	
46–60	8310 (98.85)	97 (1.15)		8388 (99.76)	20 (0.24)		8334 (99.12)	74 (0.88)		8232 (98.02)	166 (1.98)	
61–75	3139 (98.25)	56 (1.75)		3191 (99.91)	3 (0.09)		3171 (99.25)	24 (0.75)		3119 (97.74)	72 (2.26)	
75+	347 (98.86)	4 (1.14)		351 (100)	0 (0)		349 (99.43)	2 (0.57)		339 (97.69)	8 (2.31)	
Race			< 0.001			0.037			< 0.001			< 0.001
White	12,435 (98.89)	140 (1.11)		12,553 (99.83)	22 (0.17)		12,487 (99.28)	90 (0.72)		12,347 (98.31)	212 (1.69)	
AI	118 (98.33)	2 (1.67)		120 (100)	0 (0)		119 (99.17)	1 (0.83)		118 (98.33)	2 (1.67)	
API	724 (96.66)	25 (3.34)		744 (99.33)	5 (0.67)		731 (97.34)	20 (2.66)		719 (96.12)	29 (3.88)	
AA	1150 (97.62)	28 (2.38)		1177 (99.92)	1 (0.08)		1167 (99.07)	11 (0.93)		1135 (96.43)	42 (3.57)	
Unknown	85 (97.7)	2 (2.3)		87 (100)	0 (0)		87 (100)	0 (0)		86 (98.85)	1 (1.15)	
Year of Dx			0.431			0.659			0.015			0.946
2010	887 (99.11)	8 (0.89)		893 (99.78)	2 (0.22)		890 (99.44)	5 (0.56)		877 (97.99)	18 (2.01)	
2011	1374 (98.57)	20 (1.43)		1389 (99.64)	5 (0.36)		1381 (99.07)	13 (0.93)		1366 (98.13)	26 (1.87)	
2012	1748 (98.7)	23 (1.3)		1766 (99.77)	4 (0.23)		1760 (99.38)	11 (0.62)		1729 (97.85)	38 (2.15)	
2013	2186 (98.51)	33 (1.49)		2213 (99.82)	4 (0.18)		2206 (99.41)	13 (0.59)		2169 (97.83)	48 (2.17)	
2014	2529 (98.56)	37 (1.44)		2564 (99.88)	3 (0.12)		2544 (99.14)	22 (0.86)		2518 (98.21)	46 (1.79)	
2015	2718 (99.02)	27 (0.98)		2743 (99.89)	3 (0.11)		2732 (99.42)	16 (0.58)		2685 (98.03)	54 (1.97)	
2016	3070 (98.43)	49 (1.57)		3113 (99.78)	7 (0.22)		3078 (98.65)	42 (1.35)		3061 (98.2)	56 (1.8)	
Site			< 0.001			< 0.001			< 0.001			< 0.001
Gum and other mouth	301 (99.34)	2 (0.66)		302 (100)	0 (0)		299 (98.68)	4 (1.32)		295 (97.68)	7 (2.32)	
Hypopharynx	784 (98)	16 (2)		799 (99.75)	2 (0.25)		793 (98.88)	9 (1.12)		769 (95.89)	33 (4.11)	
Nasopharynx	831 (94.75)	46 (5.25)		868 (98.86)	10 (1.14)		851 (96.81)	28 (3.19)		841 (96.11)	34 (3.89)	
Oropharynx	929 (98.72)	12 (1.28)		937 (99.68)	3 (0.32)		929 (98.83)	11 (1.17)		908 (96.7)	31 (3.3)	
Other oral cavity and pharynx	283 (97.92)	6 (2.08)		289 (99.31)	2 (0.69)		286 (97.95)	6 (2.05)		286 (98.28)	5 (1.72)	
Tongue	4849 (98.86)	56 (1.14)		4901 (99.96)	2 (0.04)		4867 (99.27)	36 (0.73)		4795 (98.04)	96 (1.96)	
Tonsil	6535 (99.11)	59 (0.89)		6585 (99.86)	9 (0.14)		6566 (99.58)	28 (0.42)		6511 (98.79)	80 (1.21)	
Grade			< 0.001			0.132			< 0.001			0.264
Grade I	447 (99.78)	1 (0.22)		449 (100)	0 (0)		449 (100)	0 (0)		444 (99.11)	4 (0.89)	
Grade II	4170 (99.12)	37 (0.88)		4206 (99.9)	4 (0.1)		4193 (99.55)	19 (0.45)		4117 (98.02)	83 (1.98)	
Grade III	5902 (98.7)	78 (1.3)		5964 (99.8)	12 (0.2)		5925 (99.16)	50 (0.84)		5866 (98.19)	108 (1.81)	
Grade IV	352 (96.17)	14 (3.83)		366 (100)	0 (0)		358 (97.81)	8 (2.19)		358 (98.08)	7 (1.92)	
Unknown	3641 (98.19)	67 (1.81)		3696 (99.68)	12 (0.32)		3666 (98.79)	45 (1.21)		3620 (97.73)	84 (2.27)	
Surgery			< 0.001			0.17			< 0.001			< 0.001
Surgery	5005 (99.6)	20 (0.4)		5020 (99.92)	4 (0.08)		5017 (99.82)	9 (0.18)		4994 (99.42)	29 (0.58)	
Tumor destruction	10 (100)	0 (0)		10 (100)	0 (0)		10 (100)	0 (0)		10 (100)	0 (0)	
No	9475 (98.17)	177 (1.83)		9629 (99.75)	24 (0.25)		9543 (98.84)	112 (1.16)		9379 (97.33)	257 (2.67)	
Unknown	22 (100)	0 (0)		22 (100)	0 (0)		21 (95.45)	1 (4.55)		22 (100)	0 (0)	
Radiation			< 0.001			< 0.001			< 0.001			< 0.001
Yes	12,499 (99.08)	116 (0.92)		12,599 (99.87)	16 (0.13)		12,561 (99.56)	56 (0.44)		12,442 (98.73)	160 (1.27)	
Refused	169 (98.26)	3 (1.74)		173 (100)	0 (0)		171 (99.42)	1 (0.58)		167 (97.66)	4 (2.34)	
Unknown	1844 (95.94)	78 (4.06)		1909 (99.38)	12 (0.62)		1859 (96.62)	65 (3.38)		1796 (93.64)	122 (6.36)	
Chemotherapy			0.444			0.296			0.092			0.487
Yes	10,358 (98.61)	146 (1.39)		10,487 (99.84)	17 (0.16)		10,411 (99.09)	96 (0.91)		10,281 (98)	210 (2)	
No	4154 (98.79)	51 (1.21)		4194 (99.74)	11 (0.26)		4180 (99.38)	26 (0.62)		4124 (98.19)	76 (1.81)	
Insurance			0.485			0.457			0.318			0.081
Insured	13,750 (98.69)	183 (1.31)		13,904 (99.8)	28 (0.2)		13,822 (99.17)	115 (0.83)		13,652 (98.11)	263 (1.89)	
Uninsured	517 (98.1)	10 (1.9)		528 (100)	0 (0)		525 (99.43)	3 (0.57)		510 (96.77)	17 (3.23)	
Unknown	245 (98.39)	4 (1.61)		249 (100)	0 (0)		244 (98.39)	4 (1.61)		243 (97.59)	6 (2.41)	
Marriage			< 0.001			0.142			0.204			< 0.001
Married	8399 (99.01)	84 (0.99)		8476 (99.87)	11 (0.13)		8425 (99.27)	62 (0.73)		8349 (98.55)	123 (1.45)	
Unmarried	5385 (98.07)	106 (1.93)		5474 (99.73)	15 (0.27)		5438 (99)	55 (1)		5339 (97.3)	148 (2.7)	
Unknown	728 (99.05)	7 (0.95)		731 (99.73)	2 (0.27)		728 (99.32)	5 (0.68)		717 (97.95)	15 (2.05)	
T stage			< 0.001			< 0.001			< 0.001			< 0.001
0	62 (96.88)	2 (3.13)		62 (96.88)	2 (3.13)		63 (98.44)	1 (1.56)		63 (98.44)	1 (1.56)	
1	3467 (99.31)	24 (0.69)		3489 (99.97)	1 (0.03)		3474 (99.46)	19 (0.54)		3468 (99.4)	21 (0.6)	
2	4783 (99.25)	36 (0.75)		4814 (99.92)	4 (0.08)		4791 (99.46)	26 (0.54)		4763 (98.9)	53 (1.1)	
3	2434 (98.62)	34 (1.38)		2462 (99.84)	4 (0.16)		2447 (99.07)	23 (0.93)		2381 (96.75)	80 (3.25)	
4	2247 (97.36)	61 (2.64)		2302 (99.65)	8 (0.35)		2280 (98.74)	29 (1.26)		2215 (96.26)	86 (3.74)	
NA	331 (97.93)	7 (2.07)		338 (99.41)	2 (0.59)		333 (97.94)	7 (2.06)		335 (98.53)	5 (1.47)	
TX	1188 (97.3)	33 (2.7)		1214 (99.43)	7 (0.57)		1203 (98.61)	17 (1.39)		1180 (96.72)	40 (3.28)	
N stage			< 0.001			< 0.001			< 0.001			< 0.001
0	2341 (99.36)	15 (0.64)		2353 (99.83)	4 (0.17)		2350 (99.75)	6 (0.25)		2333 (99.19)	19 (0.81)	
1	2645 (98.55)	39 (1.45)		2671 (99.63)	10 (0.37)		2666 (99.37)	17 (0.63)		2640 (98.43)	42 (1.57)	
2	8366 (98.75)	106 (1.25)		8462 (99.88)	10 (0.12)		8405 (99.19)	69 (0.81)		8289 (97.96)	173 (2.04)	
3	733 (96.19)	29 (3.81)		762 (100)	0 (0)		745 (97.51)	19 (2.49)		717 (94.22)	44 (5.78)	
NA	331 (97.93)	7 (2.07)		338 (99.41)	2 (0.59)		333 (97.94)	7 (2.06)		335 (98.53)	5 (1.47)	
NX	96 (98.97)	1 (1.03)		95 (97.94)	2 (2.06)		92 (95.83)	4 (4.17)		91 (96.81)	3 (3.19)	

Abbreviations: AA, African American; AI, American Indian/Alaska Native; API, Asian or Pacific Islander; NA, non‐applicable; NX, unknown N stage; SD, standard deviation; TX, unknown T stage.

In terms of socioeconomic factors, uninsured patients were at a higher risk of metastasis to the bone, brain, liver, and lung compared to insured patients. Similarly, unmarried patients were associated with a higher risk of metastasis, particularly to the bone and lung. By primary site, nasopharyngeal cancer had the highest risk of all types of metastasis, followed by hypopharyngeal cancer.

### 3.2 Survival Analyses for HNC

**FIGURE 1:**
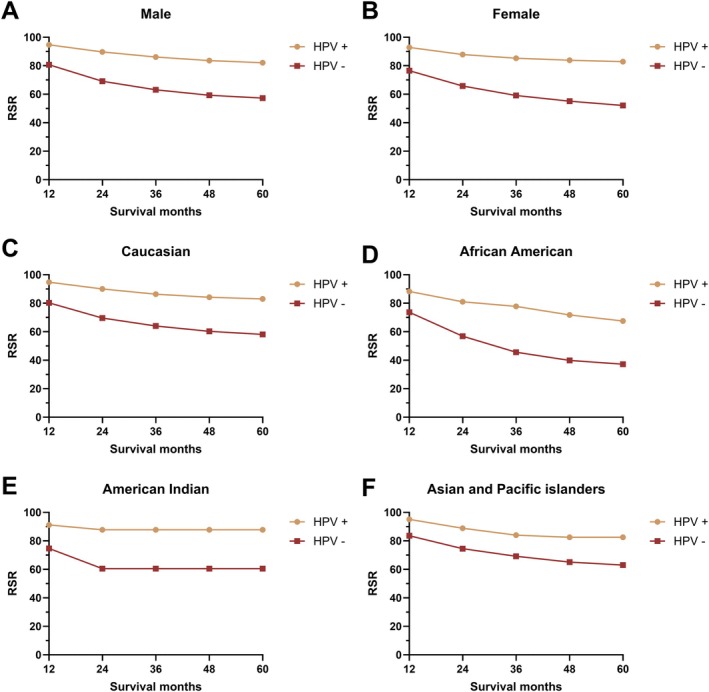
The relative survival rate of head and neck cancer by HPV status by sex (A, B) and race (C–F).

**FIGURE 2:**
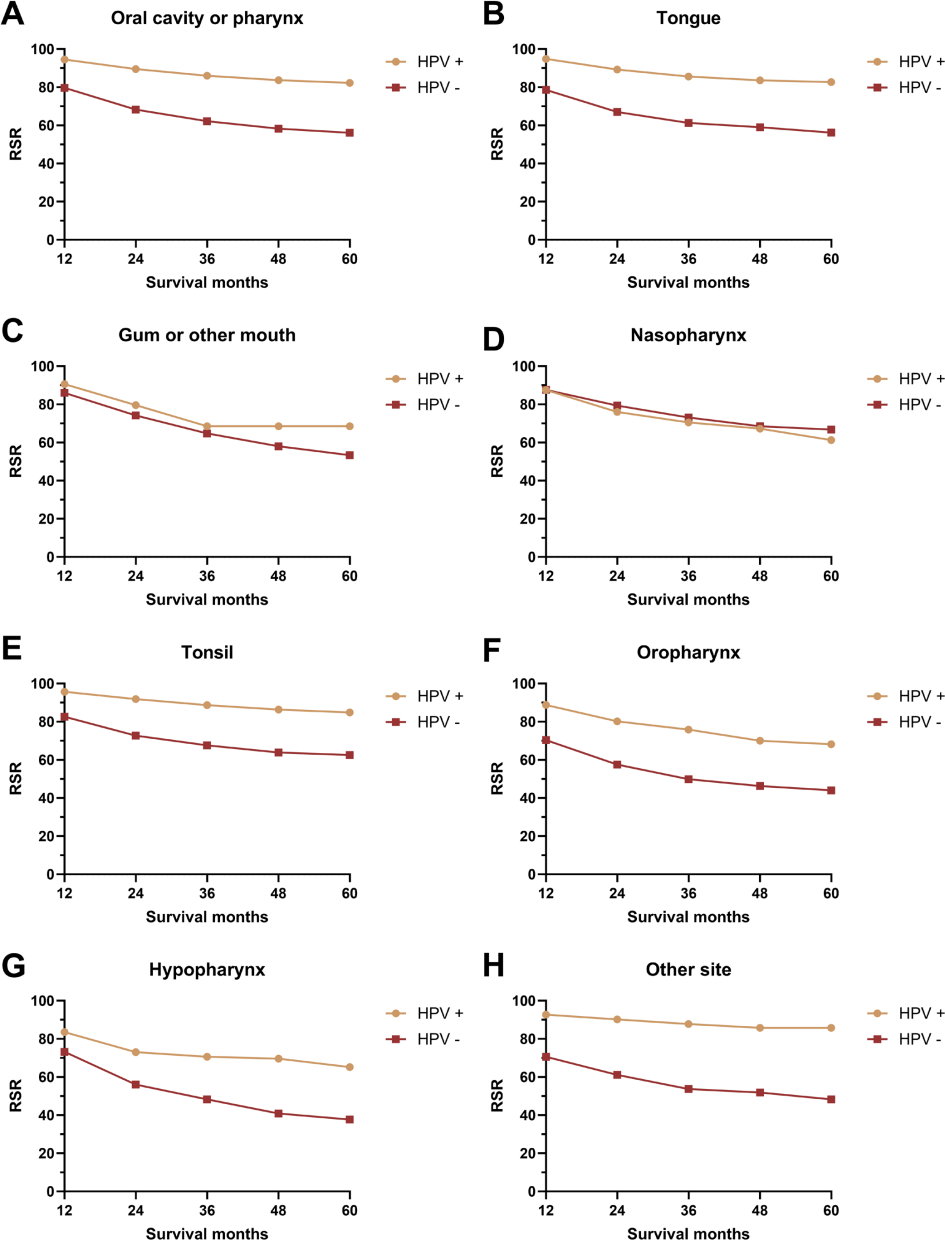
The relative survival rate of head and neck cancer by HPV status by originated site (A–H).

**FIGURE 3:**
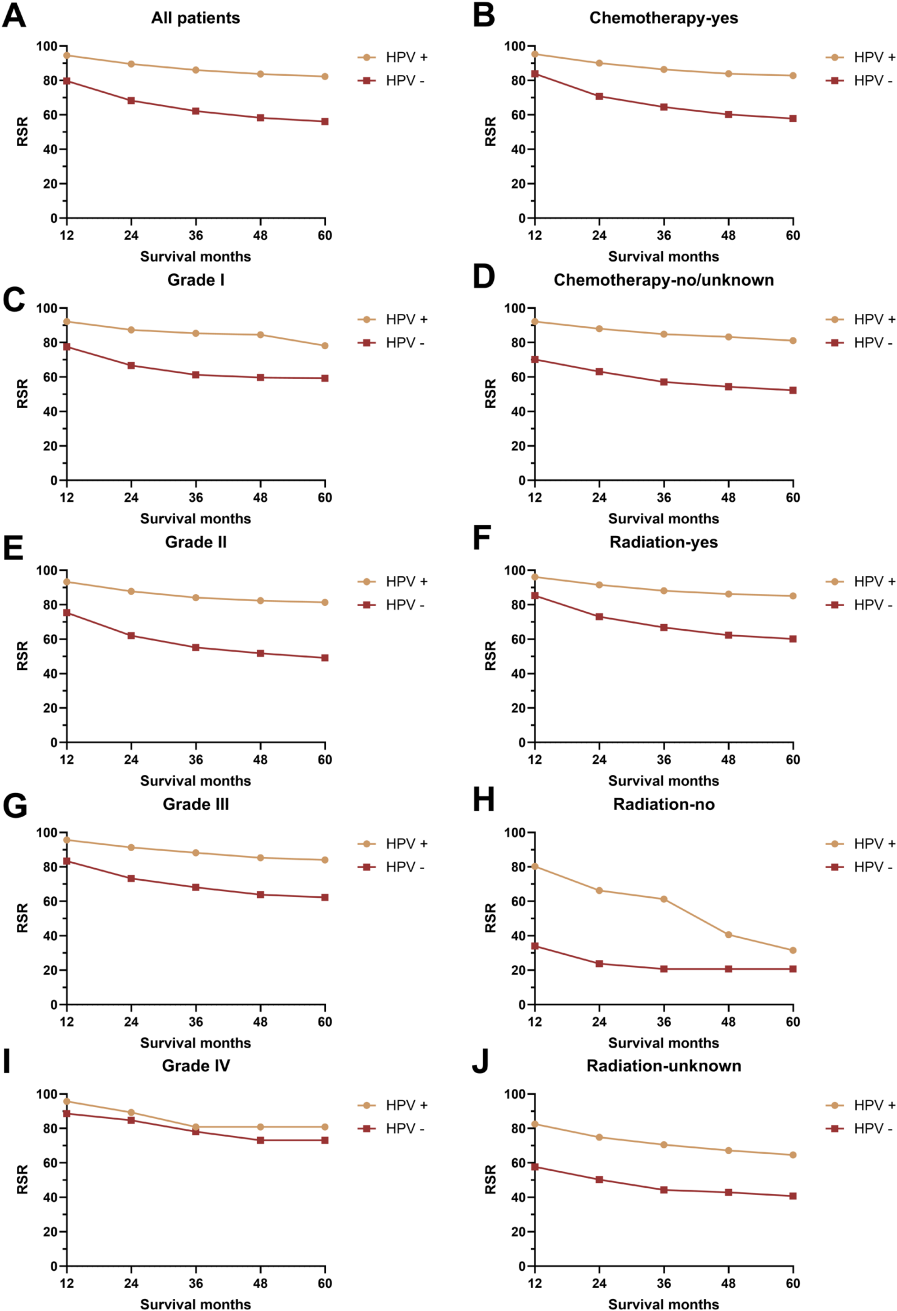
The relative survival rate of head and neck cancer by HPV status in all patients (A), by differentiation level (C, E, G, I), chemotherapy status (B, D), and radiotherapy status (F, H, J).

To further demonstrate the net prognostic impact of HPV infection on HNC patients, RSRs were calculated across clinicopathological factors. HPV infection was associated with a favorable prognosis in all patients, regardless of sex, race, and primary site, including cancers of the oral cavity, pharynx, tongue, tonsil, oropharynx, hypopharynx, and other oral cavity and pharynx regions (Figures 1, 2, 3). However, this prognosis‐stratifying effect was absent in cancers originating in the gum, other mouth regions, and nasopharynx (Figure 2C,D).

**FIGURE 4:**
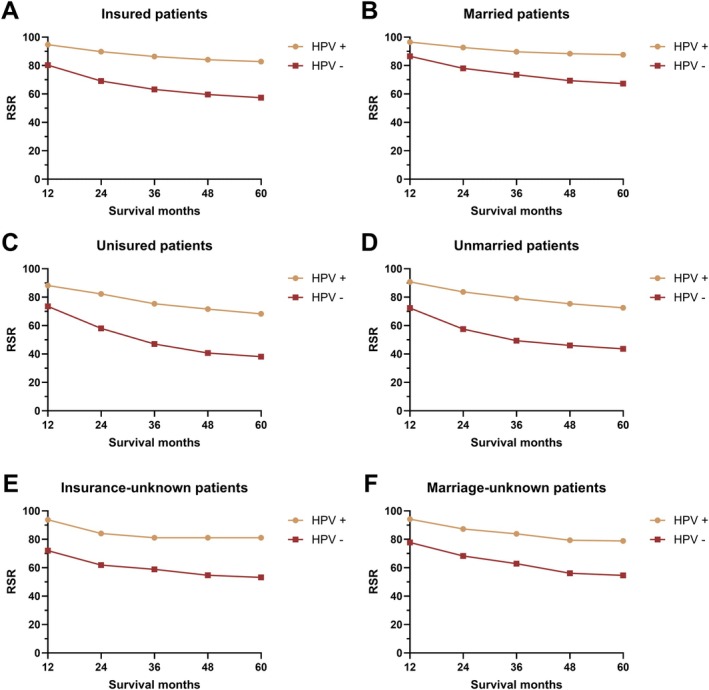
The relative survival rate of head and neck cancer by HPV status by insurance status (A, C, E) and marital status (B, D, F).

Subgroup analyses by differentiation grade revealed HPV status as a significant prognostic factor in grade I–III tumors but not in grade IV disease (Figure 3C,E,G,I). HPV status further demonstrated consistent prognostic stratification across all categories of radiation therapy, chemotherapy, insurance status, and marital status (Figures 3B,D,F,H,J and 4). To identify independent prognostic factors for HNC patients, multivariate Cox regression analyses were performed (Table 4). Significant factors associated with prognosis included race, age, year of diagnosis, primary site, differentiation grade, surgical status, radiotherapy status, chemotherapy status, insurance status, marital status, synchronous lung, brain, liver, and bone metastasis, T stage, N stage, M stage, and HPV status. Specifically, the hazard ratio (HR) for all‐cause mortality was 1.25 (1.13–1.40) for African American patients, with Caucasian patients as the reference group. HR increased with age: 1.39 (1.25–1.60) for ages 45–59, 2.26 (2.00–2.56) for ages 60–75, and 4.15 (3.46–4.98) for patients over 75 years. Patients diagnosed between 2012 and 2015 had a higher risk of death compared to those diagnosed in 2010. Hypopharyngeal and oropharyngeal cancers showed significantly higher mortality risks compared to tonsil cancer (Table 4).

**TABLE 4:** Multivariate Cox regression for head and neck patients.

Categories	Number	HR (95% CI)	*p*
All cases	14,855		
Sex			
Female	2491	Reference	
Male	164	1.00 (0.91–1.09)	> 0.05
Race			
White	12,682	Reference	
AI	121	1.24 (0.88–1.88)	> 0.05
API	760	0.87 (0.73–1.04)	> 0.05
AA	1189	1.25 (1.16–1.40)	< 0.0001
Unknown	103	0.23 (0.10–0.57)	< 0.001
Age group			
0–44	2779	Reference	
45–59	8479	1.39 (1.25–1.56)	< 0.0001
60–75	3236	2.26 (2.00–2.56)	< 0.0001
75+	361	4.15 (3.46–4.98)	< 0.0001
Year of diagnosis			
2010	901	Reference	
2011	1414	1.09 (0.94–1.28)	> 0.05
2012	1788	1.19 (1.02–1.39)	< 0.01
2013	2240	1.35 (1.16–1.57)	< 0.0001
2014	2585	1.31 (1.13–1.53)	< 0.0001
2015	2765	1.31 (1.11–1.54)	< 0.001
2016	3162	1.04 (0.85–1.28)	> 0.05
Site			
Tonsil	6650	Reference	
Gum and other mouth	307	0.99 (0.80–1.23)	> 0.05
Hypopharynx	811	1.36 (1.19–1.55)	< 0.0001
Nasopharynx	890	0.98 (0.82–1.17)	> 0.05
Oropharynx	955	1.40 (1.23–1.60)	< 0.0001
Other oral cavity and pharynx	307	1.33 (0.49–3.67)	> 0.05
Tongue	4935	1.01 (0.92–1.10)	> 0.05
Tonsil	6650	Reference	
Differentiation			
Grade I	452	Reference	
Grade II	4254	1.08 (0.89–1.31)	> 0.05
Grade III	6033	0.81 (0.67–0.99)	< 0.01
Grade IV	370	0.71 (0.52–0.99)	< 0.01
Unknown	3746	0.86 (0.70–1.05)	> 0.05
Surgery			
Surgery	5047	Reference	
Tumor destruction	10	2.82 (0.70–11.33)	> 0.05
NO	9765	1.92 (1.74–2.12)	< 0.0001
Unknown	33	2.46 (1.25–4.86)	< 0.001
Radiotherapy			
Yes	12,685	Reference	
Refused	176	3.76 (2.98–4.73)	< 0.0001
Unknown	1994	2.33 (2.12–2.57)	< 0.0001
Chemotherapy			
Yes	10,560	Reference	
No	4295	1.46 (1.33–1.60)	< 0.0001
Insurance			
Insured	14,049	Reference	
Uninsured	529	1.33 (1.14–1.55)	< 0.0001
Unknown	277	0.92 (0.71–1.18)	> 0.05
Marriage			
Married	8539	Reference	
Unmarried	5544	1.60 (1.48–1.73)	< 0.0001
Unknown	772	1.20 (1.02–1.41)	< 0.01
Lung metastasis			
No	14,405	Reference	
Yes	286	1.53 (1.23–1.91)	< 0.0001
NA	2	149,055.48 (0.00–0.00)	> 0.05
Unknown	162	1.64 (0.92–2.90)	> 0.05
Brain metastasis			
No	14,681	Reference	
Yes	28	2.71 (1.70–4.30)	< 0.0001
NA	2	NA	> 0.05
Unknown	144	2.37 (0.77–7.27)	> 0.05
Liver metastasis			
No	14,591	Reference	
Yes	122	1.34 (1.02–1.75)	< 0.01
NA	2	NA	> 0.05
Unknown	140	0.13 (0.04–0.41)	< 0.0001
Bone metastasis			
No	14,512	Reference	
Yes	197	1.52 (1.20–1.92)	< 0.0001
NA	3	0.00 (0.00–0.00)	> 0.05
Unknown	143	2.40 (1.03–5.58)	< 0.01
T stage			
0	64	Reference	
1	3502	0.99 (0.55–1.79)	> 0.05
2	4844	1.45 (0.81–2.60)	> 0.05
3	2473	2.29 (1.28–4.11)	< 0.001
4	20	3.38 (1.89–6.06)	< 0.0001
NA	365	1.96 (0.62–6.17)	> 0.05
TX	1287	1.50 (0.83–2.70)	> 0.05
N stage			
0	66	Reference	
1	2701	1.33 (1.17–1.51)	< 0.0001
2	8505	1.51 (1.35–1.68)	< 0.0001
3	767	2.17 (1.85–2.55)	< 0.0001
NA	365	NA	> 0.05
NX	151	1.21 (0.83–1.74)	> 0.05
M stage			
0	13,894	Reference	
1	596	1.79 (1.46–2.20)	< 0.0001
NA	365	NA	> 0.05
HPV status			
Negative	4727	Reference	
Positive	10,128	0.50 (0.46–0.54)	< 0.0001

Abbreviations: AA, African American; AI, American Indian/Alaska Native; API, Asian or Pacific Islander; NA, non‐applicable; NX, unknown N stage; SD, standard deviation; TX, unknown T stage.

Grade III and IV tumors were associated with lower mortality risks, with HRs of 0.81 (0.67–0.99) and 0.71 (0.52–0.99), respectively. Patients without surgery had a higher risk of death (HR = 1.92 (1.74–2.12)) compared to those who underwent surgery. Refusal of radiotherapy or chemotherapy was linked to significantly higher risks of death (HR = 3.76 (2.98–4.73) and HR = 1.46 (1.33–1.60), respectively). Uninsured and unmarried patients had elevated mortality risks (HR = 1.33 (1.14–1.55) and HR = 1.60 (1.48–1.73), respectively) compared to their insured or married counterparts. Synchronous metastases to the lung, brain, liver, and bone were also associated with higher risks of death, with HRs of 1.53 (1.23–1.91), 2.706 (1.70–4.30), 1.34 (1.02–1.75), and 1.52 (1.20–1.92), respectively, compared to non‐metastatic patients. Advanced T, N, and M stages were linked to increased mortality risk. Positive HPV status was associated with a significantly lower risk of death (HR = 0.50 (0.46–0.54)) compared to HPV‐negative patients.

To further clarify HPV's role in HNC, propensity score matching was used to match cases by HPV status, followed by univariate Cox regression analyses in various subgroups (Table 5). Consistently lower mortality risks in HPV‐positive patients were observed across most subgroups, confirming the favorable prognostic role of HPV in HNC cases.

**TABLE 5:** Univariate Cox regression analyses of HPV status in different categories in matched cases.

Categories	Number	HR (95% CI)	*p*
Sex			
Female	1466	0.52 (0.42–0.63)	< 0.001
Male	5634	0.60 (0.54–0.66)	< 0.001
Age			
0–45	1335	0.53 (0.41–0.68)	< 0.001
46–60	3830	0.54 (0.47–0.62)	< 0.001
61–75	1723	0.62 (0.53–0.74)	< 0.001
75+	212	0.83 (0.58–1.20)	> 0.05
Race			
White	5799	0.58 (0.52–0.64)	< 0.001
AI	70	0.65 (0.25–1.72)	> 0.05
API	430	0.55 (0.36–0.84)	< 0.01
AA	750	0.55 (0.43–0.71)	< 0.001
Unknown	51	1.05 (0.07–16.87)	> 0.05
Year of diagnosis			
2010	516	0.64 (0.48–0.86)	< 0.001
2011	813	0.60 (0.48–0.77)	< 0.001
2012	944	0.54 (0.43–0.67)	< 0.001
2013	1096	0.59 (0.48–0.73)	< 0.001
2014	1194	0.54 (0.44–0.67)	< 0.001
2015	1176	0.68 (0.52–0.87)	< 0.001
2016	1361	0.45 (0.31–0.67)	< 0.001
Site			
Gum and other mouth	148	0.84 (0.43–1.64)	> 0.05
Hypopharynx	425	0.66 (0.47–0.93)	< 0.05
Nasopharynx	556	1.22 (0.85–1.75)	> 0.05
Oropharynx	632	0.69 (0.53–0.91)	< 0.01
Other oral cavity and pharynx	216	0.37 (0.21–0.66)	< 0.001
Tongue	2319	0.55 (0.47–0.64)	< 0.001
Tonsil	2804	0.50 (0.42–0.58)	< 0.001
Differentiation			
Grade I	295	0.53 (0.32–0.87)	< 0.05
Grade II	2378	0.53 (0.45–0.62)	< 0.001
Grade III	2582	0.64 (0.54–0.75)	< 0.001
Grade IV	205	0.82 (0.44–1.53)	> 0.05
Unknown	1640	0.57 (0.47–0.69)	< 0.001
Surgery			
Surgery	2177	0.54 (0.43–0.67)	< 0.001
Tumor destruction	9	1.00 (0.06–15.99)	> 0.05
NO	4892	0.59 (0.53–0.65)	< 0.001
Unknown	22	0.87 (0.12–6.21)	> 0.05
Radiotherapy			
Yes	5829	0.54 (0.49–0.61)	< 0.001
Refused	96	0.43 (0.25–0.75)	< 0.01
Unknown	1175	0.65 (0.54–0.78)	< 0.001
Chemotherapy			
Yes	4906	0.59 (0.53–0.66)	< 0.001
No	2194	0.55 (0.47–0.65)	< 0.001
Insurance			
Insured	6612	0.59 (0.53–0.65)	< 0.001
Uninsured	318	0.46 (0.24–0.85)	< 0.001
Unknown	170	0.54 (0.38–0.77)	< 0.05
Marriage			
Married	3656	0.57 (0.49–0.66)	< 0.001
Unmarried	3021	0.54 (0.48–0.61)	< 0.001
Unknown	423	0.73 (0.50–1.07)	> 0.05
Lung metastasis			
NA	1	0.79 (0.55–1.13)	> 0.05
No	6822	0.56 (0.51–0.62)	< 0.001
Unknown	104	0.67 (0.35–1.30)	> 0.05
Yes	173	0.79 (0.55–1.13)	> 0.05
Brain metastasis			
NA	1	1.35 (0.45–4.01)	> 0.05
No	6983	0.58 (0.53–0.63)	< 0.001
Unknown	95	0.72 (0.36–1.45)	> 0.05
Yes	21	1.35 (0.45–4.01)	> 0.05
Liver metastasis			
NA	1	0.90 (0.50–1.59)	> 0.05
No	6932	0.57 (0.52–0.63)	< 0.001
Unknown	90	0.60 (0.28–1.28)	> 0.05
Yes	77	0.90 (0.50–1.59)	> 0.05
Bone metastasis			
NA	1	0.80 (0.52–1.22)	> 0.05
No	6882	0.58 (0.52–0.63)	< 0.001
Unknown	93	0.64 (0.31–1.32)	> 0.05
Yes	124	0.80 (0.52–1.22)	> 0.05
T stage			
0	26	1.25 (0.23–6.86)	> 0.05
1	1437	0.63 (0.47–0.84)	< 0.01
2	2031	0.48 (0.39–0.60)	< 0.001
3	1313	0.54 (0.44–0.65)	< 0.001
4	1351	0.67 (0.57–0.78)	< 0.001
NA	251	0.38 (0.21–0.67)	< 0.001
TX	691	0.50 (0.37–0.68)	< 0.001
N stage			
0	1347	0.54 (0.42–0.69)	< 0.001
1	1461	0.61 (0.49–0.76)	< 0.001
2	3542	0.60 (0.53–0.68)	< 0.001
3	407	0.57 (0.41–0.80)	< 0.001
NA	251	0.38 (0.21–0.67)	< 0.001
NX	92	0.82 (0.38–1.76)	> 0.05
M stage			
0	6482	0.56 (0.50–0.62)	< 0.001
1	367	0.74 (0.58–0.96)	< 0.05
NA	251	0.38 (0.21–0.67)	< 0.001

Abbreviations: AA, African American; AI, American Indian/Alaska Native; API, Asian or Pacific Islander; NA, non‐applicable; NX, unknown N stage; SD, standard deviation; TX, unknown T stage.

## 4 Discussion

The current study demonstrated the clinical characteristics of HNC based on HPV status, primary site, and metastatic patterns, as well as the survival implications of HPV in HNC. To the best of our knowledge, this is the first population‐based study providing representative and generalizable data. The novelty and impact of this study lie in its comprehensive analysis of HNC epidemiology using a population‐based dataset. To further mitigate confounding biases when elaborating on the prognostic role of HPV, we implemented propensity score matching, enhancing the robustness of our findings.

HNC represents a heterogeneous group of diseases, with 75% associated with smoking and alcohol exposure, and 25% related to HPV infection [8, 46]. HPV‐positive and HPV‐negative HNC represent two distinct disease types in terms of natural history and clinicopathological characteristics. HPV‐infected patients tend to be younger than their non‐infected counterparts, suggesting that HPV may accelerate the carcinogenesis process. Furthermore, HNC patients with HPV infection are less likely to have tobacco or alcohol exposure, indicating that HPV can promote carcinogenesis independently of these factors [30]. Most cancers originating from the gum, hypopharynx, and nasopharynx are HPV‐negative, whereas the majority of tongue and tonsil cancers are HPV‐positive, suggesting differences in the etiology of various HNC types. The higher HPV positivity among Caucasian patients (89.6%) may be attributed to the higher prevalence of HPV infection in these populations (71.5%) compared to African Americans (66.2%) [32]. The association between socioeconomic status (e.g., income, education), race, tobacco exposure, sexual behavior, and HPV prevalence in HNC may be explained through multifactorial mechanisms [47]. Despite the more advanced N stage in HPV‐positive HNC patients, these patients exhibit a lower incidence of M1 disease and metastasis to the lung, brain, liver, or bone, though the underlying mechanism remains unknown. The prognostic value of HPV infection is limited in cancers originating from the gum, other mouth tissues, and nasopharynx, as revealed by both RSRs and Cox regression analyses after propensity score matching. Although the specific molecular events driving the better prognosis in HPV‐infected patients remain unclear, there is a growing trend toward advocating de‐intensified treatment to reduce post‐treatment toxicity without compromising cancer control. The eighth edition of the AJCC staging system introduces a unique classification for HPV‐positive squamous oropharyngeal cancer, resulting in downstaging for 92% of cases and increasing the proportion of stage I patients from 3% to 64% [48]. However, the recommended treatment protocol by stage based on this novel staging system has not yet been published. In line with the modification of the staging system, de‐intensified treatment strategies are under exploration, particularly for HPV‐positive patients with a lower risk of recurrence, such as those with a smoking history of less than 10 pack‐years and non‐T4 disease [17, 49, 50, 51, 52]. However, robust evidence supporting de‐escalation remains lacking to date. The clinical community expects multicenter randomized controlled trials to clarify which patients are most likely to benefit from such strategies. The baseline characteristics of patients in the current study differ slightly from those in another study based on the NCDB, which can be attributed to the differing inclusion criteria [53]. Regarding the clinical testing of HPV, a recent study highlights critical limitations of relying solely on p16 as a surrogate for HPV status in oropharyngeal cancer [54]. The notable discordance between p16 and HPV nucleic acid status—particularly in regions with low HPV‐attributable fractions—underscores the prognostic heterogeneity masked by p16 alone. Patients with discordant results (p16+/HPV− or p16−/HPV+) exhibited intermediate survival outcomes, distinct from both HPV‐driven and HPV‐negative tumors. These findings advocate for mandatory dual testing where HPV status informs therapeutic decisions, ensuring accurate risk stratification and tailored treatment, especially in low‐HPV‐prevalence populations. In the multivariate Cox regression, we observed counterintuitive hazard ratios for grade and year of diagnosis. This phenomenon may be attributed to residual confounding, where unmeasured or inadequately adjusted covariates exhibit stronger prognostic associations than tumor grade and year of diagnosis. Such paradoxical findings are consistent with a prior report [55]. In terms of metastasis in HNC, the current study primarily focuses on synchronous metastasis, which may provide valuable data for clinical screening at initial diagnosis. Historical studies published in the 1990s reported metastasis rates of 10%–17% in HNC, a figure notably higher than that observed in our study [56, 57, 58]. This counterintuitively higher incidence of metastasis in earlier studies, despite less‐developed methods for detecting metastasis, may be attributed to delayed diagnosis at that time. Different cancer types exhibit varying metastatic potentials, likely influenced by intrinsic tumor biology. For instance, while oropharyngeal cancer generally presents with a more advanced N stage compared to hypopharyngeal cancer, the incidence of metastasis is higher in hypopharyngeal cancer (6.8% vs. 5.7%). The lungs are the most common site of metachronous metastasis in HNC, followed by bone and liver, consistent with previous publications [59, 60]. Advanced stage grouping, increasing N classification, node positivity, extranodal extension, and HPV negativity are factors associated with the development of distant metastases, independent of any changes in treatment paradigms [23]. Thus, the most effective way to reduce the incidence of distant metastases is to diagnose HNSCC at earlier stages [23]. Nasopharyngeal cancer had the highest percentage of M1 disease (9.9%), followed by hypopharyngeal cancer (6.8%), consistent with a previous study based on the National Cancer Data Base in the USA [53].

Despite novel findings, the current study should be interpreted in the context of its limitations. First, only patients with definite HPV status were included, as the percentages of unknown HPV status were 77.16%, 65.51%, 56.28%, 49.50%, 43.80%, 39.79%, and 33.25% across calendar years. As a result, the epidemiological trends reported here may be biased, particularly in the earlier periods with limited patient enrollment. Due to incomplete HPV information, HPV‐based incidence of HNC could not be reported. However, to the best of our knowledge, SEER data remain the most comprehensive publicly available resource, and future studies with more detailed data may provide further insights into this topic. Second, HPV subtypes were not available in the current dataset, preventing comparisons based on HPV subtypes. Third, caution should be exercised when applying these findings to predict epidemiological trends outside the USA. Fourth, this SEER‐based study may be subject to bias due to potential misclassification or under‐registration in the SEER database.

In conclusion, the current study, based on a large population‐based database, provides generalizable epidemiological data on HNC in terms of HPV status, primary site, and metastatic patterns, as well as the prognostic impact of HPV infection. Analyzing the baseline characteristics of HNC enhances our understanding of the disparities among different HNC subtypes, which may help tailor clinical decision‐making and improve the design of clinical trials for each subtype. Furthermore, clarifying the prognostic role of HPV in HNC may reveal the epidemiological link between HNC pathogenesis and HPV infection, facilitating further bench studies on the role of HPV in HNC.

## Author Contributions

Study design: Kangwen Guo and Xiaoqiong Yi. Data collection: Kangwen Guo, Jinmei Li, Haiyin Ye, and Xiaoqiong Yi. Statistical analyses: Kangwen Guo, Jinmei Li, Haiyin Ye, and Xiaoqiong Yi. Manuscript drafting: Kangwen Guo, Haiyin Ye, and Jinmei Li. Manuscript revision: Kangwen Guo and Xiaoqiong Yi.

## Ethics Statement

This retrospective study utilized fully anonymized, pre‐existing data from the SEER database, with no identifiable patient information. Ethical approval was waived in accordance with national regulations and the Declaration of Helsinki, which exempts studies involving anonymized data from formal ethics review when the research poses no risk to participants.

## Conflicts of Interest

The authors declare no conflicts of interest.

## Data Availability

All the data that support the results of the current study are publicly available in the SEER database (https://seer.cancer.gov/).
